# Improving Stress Management and Sleep Hygiene in Intelligent Homes

**DOI:** 10.3390/s21072398

**Published:** 2021-03-30

**Authors:** Asterios Leonidis, Maria Korozi, Eirini Sykianaki, Eleni Tsolakou, Vasilios Kouroumalis, Danai Ioannidi, Andreas Stavridakis, Margherita Antona, Constantine Stephanidis

**Affiliations:** 1 Institute of Computer Science, Foundation for Research and Technology—Hellas (FORTH), 70013 Heraklion, Crete, Greece; korozi@ics.forth.gr (M.K.); eirinisi@ics.forth.gr (E.S.); tsolakou@ics.forth.gr (E.T.); vic@ics.forth.gr (V.K.); ioanidi@ics.forth.gr (D.I.); astavr@ics.forth.gr (A.S.); antona@ics.forth.gr (M.A.); cs@ics.forth.gr (C.S.); 2 Department of Computer Science Heraklion, University of Crete, 70013 Heraklion, Crete, Greece

**Keywords:** ambient intelligence, smart home, stress management, sleep hygiene, sleep monitoring, contextual awareness

## Abstract

High stress levels and sleep deprivation may cause several mental or physical health issues, such as depression, impaired memory, decreased motivation, obesity, etc. The COVID-19 pandemic has produced unprecedented changes in our lives, generating significant stress, and worries about health, social isolation, employment, and finances. To this end, nowadays more than ever, it is crucial to deliver solutions that can help people to manage and control their stress, as well as to reduce sleep disturbances, so as to improve their health and overall quality of life. Technology, and in particular Ambient Intelligence Environments, can help towards that direction, when considering that they are able to understand the needs of their users, identify their behavior, learn their preferences, and act and react in their interest. This work presents two systems that have been designed and developed in the context of an Intelligent Home, namely CaLmi and HypnOS, which aim to assist users that struggle with stress and poor sleep quality, respectively. Both of the systems rely on real-time data collected by wearable devices, as well as contextual information retrieved from the ambient facilities of the Intelligent Home, so as to offer appropriate pervasive relaxation programs (CaLmi) or provide personalized insights regarding sleep hygiene (HypnOS) to the residents. This article will describe the design process that was followed, the functionality of both systems, the results of the user studies that were conducted for the evaluation of their end-user applications, and a discussion about future plans.

## 1. Introduction

In the case of domestic life, the advancement of the Internet of Things (IoT) [1] in combination with cloud computing [2] has led to the emergence of the Intelligent Home (or Smart Home) concept, which is one of the most prevalent environments enriched with Ambient Intelligence (AmI) [3] technologies. An Intelligent Home is an environment that is equipped with sophisticated mechanisms (e.g., activity recognition, user tracking, and user profiling) and various household artifacts (e.g., bed, bedside table, closet, and sofa), which are technologically enhanced so as to gather information regarding their usage, and, in some cases, even to act independently without the need of human intervention [4].

In a world where technology is omnipresent, the question arises as to how its role towards enhancing well-being can be optimized. For example, the concept of positive computing aims to develop technologies to support well-being and human potential for anyone through preventative mental health support, strengthening mental health through positive behavior change and self-reflection, and through increasing empathy towards, and awareness of, mental health [5].

Towards this direction, it needs to be investigated how Intelligent Homes can support their residents both from a physical and psychological perspective, so as to help them achieve a high level of wellbeing and a good quality of life, especially during uneasy times, such as the COVID-19 pandemic. In particular, during this period social isolation, economic instability, and the overarching feeling of loss (loss of income, routine, or social interaction), has led to increased stress levels and has caused sleep disturbances [6].

In today’s fast-paced and complex society, increasing amounts of people are suffering from stress-related problems and poor sleep quality. High stress levels, together with the endocrine response to stress, can influence disease risk and lead to physical and mental disorders [7]. Sleep, on the other hand, is also essential for optimal cognitive performance, physiological processes, emotional regulation, and overall well-being. To this end, it is important to keep stress under control, regulate the sleep process, and improve the overall sleep quality. Intelligent Environments provide the tools and appropriate infrastructure to accomplish the above by building user-centric, context-aware, non-intrusive systems [8].

Towards this direction, this work presents CaLmi and HypnOS, two systems that utilize the infrastructure of the Intelligent Home of ICS-FORTH (http://ami.ics.forth.gr/ accessed on 3 February 2021), so as to retrieve and extract relevant contextual information regarding the user’s daily routines and habits (e.g., diet, work schedule, and fitness), and act accordingly towards relaxing the residents and improving their sleep quality. In particular, CaLmi is a stress management system that uses both biofeedback and contextual information in order to detect whether the stress level of an individual increases, and it outline potential causes for that increase. It offers a pervasive relaxation program player that can create a relaxing experience by projecting multimedia to all room’s display areas, playing appropriate sound(s) from the room’s speakers, adjusting the ambient lighting conditions, releasing a pleasant scent into the room, etc. HypnOS, on the other hand, is an unobtrusive sleep monitoring and recommendation system that not only detects sleep abnormalities, but it also presents insights to the residents about the potential causes of their sleep-related issues, enabling them to act accordingly. HypnOS also interoperates with the pervasive relaxation program player of CaLmi in order to help users fall asleep when required.

This article presents: (i) a discussion of background theory and related work, (ii) the design process that was followed, (iii) the functionality of the CaLmi and HypnOS systems, (v) the results of the user studies that were conducted for the evaluation of their end-user applications, and (iv) a discussion about future plans.

## 2. Background Theory

### 2.1. Implications of Stress in Everyday Life

Stress is a term of everyday life that everyone uses when feeling nervous, threatened, or overwhelmed, and it can be defined as the body’s response to demanding situations [9]. Acute (short-term) stress is “a strong physiological response to a novel and unpredictable threatening event” [10] and it is actually good for maintaining human survival and prosperity by increasing alertness in dangerous (e.g., escape from a lion) or demanding situations (e.g., complete a project by a certain date). However, if acute stress is repeatedly experienced (episodic acute stress) without adequate relaxation periods in between, it can become maladaptive [11] and turn into chronic (long-term) stress, which can have a serious impact on physical (e.g., is associated with the metabolic syndrome, which is a cluster of risk factors that increase the chance of developing heart disease and type 2 diabetes [12]) and mental (e.g., depression [13]) health. Furthermore, besides adverse health effects, information processing (particularly memory and attention) [14] and decision making [15] skills can be decelerated by bad stress.

Nowadays, more and more people are suffering from stress-related problems [16], which might cause them, among others, a lack of sleep (27%) and lack of interest in activities (26%), unhealthy eating habits (25%), pre-occupation with their problems (18%), and unwillingness to leave the house (16%). Those behavioral changes, together with the endocrine response to stress, can influence disease risk and lead to physical and mental disorders [7]. Accordingly, it is important to monitor the stress levels and keep them under control when necessary.

### 2.2. Stress Monitoring Techniques

There is not one particular indicator that reveals increased levels of stress. However, stress causes several changes in the human body and mind and, thus, it is possible to be detected. In order to achieve stress detection, subjective questionnaire data, body and speech tracking data, physiological signals, and contextual information are collected and analyzed.

Many researchers have elaborated expert questionnaires (e.g., the Perceived Stress Scale-PSS [17] and the Standard Stress Scale-SSS [18]) as a stress diagnostic tool. This approach requires time, effort, and mental health awareness from the user, since it lacks automatic detection and constantly asks for user input. Given the latest advancements in the domain of Ambient Intelligence and the proliferation of Intelligent Environments, this process could be lightened by automatically detecting cues indicating stress—without directly asking the user. If necessary, the system could intervene to ask for a confirmation in order to validate its decisions.

Speech spectral analysis and analysis of lexical choices [19], facial expressions, gestures [20], and iris and pupil attributes [21] have also been tested. A combination of these methods can deliver quite accurate results, however tracking of the relevant parameters cannot be achieved in real-life situations (e.g., while on the move) and the reactions, which are based on the somatic nervous system, can be faked [22].

On the other hand, physiological signals can be unobtrusively measured using wearable devices, thus they are more applicable for constantly tracking people during their daily routines and activities. Blood pressure (BP), heart rate (HR) and its variability (HRV) [23], electrodermal activity (EDA) [24], skin temperature (ST) [25], and breathing rate (BR) [26] are the mostly used physiological signals for stress recognition. Electroencephalogram (EEG) [27] and hormonal signals, such as Salivary Cortisol (SC) [28], are also great choices, but they cannot be measured using a wearable device. EDA is not affected by the parasympathetic nervous system (as most physiological signals do) and. for this reason, it is considered by many as the best choice for stress recognition [29]. EDA peaks indicate an emotional arousal, which, however, is not always stress related [30].

By correctly interpreting contextual information, additional knowledge regarding a persons’ levels of stress can be extracted [31]. Context-based stress detection is possible by identifying stressful life events (e.g., unexpected health problems [32]) and behavioral stress responses (e.g., increased smoking behaviors [33], overeating or undereating [34], and increased alcohol consumption [35]).

### 2.3. Stress Reduction Methods

Many different calming strategies have been examined for several years. There may be differences in how each person experiences recovery from stress through specific activities, but several relaxation techniques have been shown to reduce arousal [36].

Research has shown that visual exposure to slides [37], images [38,39], or videos [40,41] of nature scenes reduces stress and leads to higher levels of positive affect in comparison with urban scenes. Additionally, auditory stimulation (e.g., sounds of running water and birdsong) offers efficient recovery after a stressor [42]. Essential oils can be used in the space to create smells of nature that stimulate the olfactory system. Hinoki cypress leaf oil [43] and lavender aroma [44] are some of the scents that promote relaxation. By combining those visual, auditory, and olfactory stimulations, a multimodal relaxing atmosphere can be created indoors.

Mindfulness-based stress reduction (MBSR) has also been used to reduce stress and improve and maintain mental well-being in a wide range of people with significant differences, since it can be adaptable to specific needs [45,46,47,48,49,50]. The most commonly used mindfulness practices are body scan, sitting meditation, and mindful hatha yoga [51]. Progressive muscle relaxation [52], diaphragmatic breathing [53], and guided imaginary [54] are additional mindfulness practices that can be used to reduce stress. Nevertheless, a large number of people struggle to quiet mental chatter and, thus, to be mindful. Therefore, continuous practice and effort is needed to be able to calm oneself with MBSR [55].

Researchers [56,57] have also observed that music, alone or as part of a relaxation technique, plays an important role in reducing stress in various settings. Generally, listening to music prior to stress boosts ANS recovery afterward [58], and listening to relaxing music after stress leads to quicker recovery in comparison with silence [59]. There are also several studies that compare the effects of different types of music on the relaxation response. Classical music appears to be more beneficial for stress management when compared to other music types (e.g., hard rock [60], heavy metal [61], and jazz and pop [62]). Furthermore, instrumental music accompanied by nature sounds seems to be another great choice [63].

Writing can also be a preventative and self-help tool for stress reduction. Nevertheless, it is important to keep in mind that expressive writing does not provide immediate stress relief. Writing down unpleasant experiences might be painful at the present time, but it provides long-term improvements in mood and reductions in stress levels [64,65].

Creating a relaxing space around people when they are practicing a relaxation technique can improve the effectiveness of that technique. Besides ambient temperature (i.e., moderately warm conditions lead to relaxation [66]), by properly changing the ambient lighting and scent settings, which are easily adjustable, the atmosphere of a room can be perceived as relaxing and have a positive effect on the mood. More specifically, peripheral and non-uniform spatial lighting with low brightness helps to create a relaxing atmosphere [67]. Cool colors (e.g., blue, green, purple) have been associated with peaceful, calm, and restful environments [68].and aromatherapy, which uses essential oils for activating the olfactory system, is used as a complementary therapy for stress relief [69].

Intelligent Environments can provide a variety of ambient facilities (e.g., pervasive displays to present multimedia, smart lighting systems, ambient sound players, odor diffusers, etc.) to their residents and, thus, with the appropriate design, conditions that meet the above relaxation techniques can be created.

### 2.4. Implications of Poor Sleep Quality in Everyday Life

These days, people have come to consider sleeping as a luxury instead of a necessity. Hours that should be spent resting are instead spent on television, games, the Internet, and work. According to the National Sleep Foundation’s updated sleep duration recommendations [70], the proposed sleep duration is 7–9 h for young adults and adults, and 7–8 h for older adults. Unfortunately, 51% of the adults globally reported that they are unsatisfied with their sleep while fewer people in 2020 are interested in improving their sleep compared to 2019, according to a recent survey that was conducted by Philips [71].

Overall, sleep is a significant component of physical and mental health, as well as of overall well-being. Short sleep duration as well as sleep disturbances are associated with poor performance of the body’s daily tasks, depressive disorders, impaired memory, decreased motivation, obesity, and even cardiac morbidity, as is strongly indicated by many experimental studies [72,73,74,75,76,77]. Consequently, it is important to regulate the sleep process and improve the overall sleep quality. There is the need to monitor sleep behavior so as to identify sleep patterns, and to provide effective guidance to improve sleep quality, in order to achieve this purpose and improve quality of life.

### 2.5. Sleep Monitoring Techniques

In the past years, assessing sleep quality has gained considerable attention, especially in sleep medicine. Towards this effort, different methods have been developed, including objective methods and subjective measures.

Objective methods provide more detailed information on sleep architecture and clinical diagnoses. Moreover, they are useful for the objective assessment of daytime sleepiness and the documentation of specific sleep behaviors and patterns [78]. The “gold standard” of objective methods is polysomnography [79], which describes the recording, analysis, and interpretation of multiple, simultaneous physiologic characteristics during sleep. It is a complex procedure, because it requires lots of special equipment and trained personnel to setup the equipment [80]. Actigraphy is another laboratory test for assessing sleep disorders objectively. Actigraphs are battery powered, long-life, light-weight, non-invasive, wearable accelerometers that record acceleration and deceleration of body movements, which indirectly indicates the state of sleep or wakefulness [81]. They are used in clinical settings to sense basic sleep patterns, such as hours slept and the number of awakenings [78]. The aforementioned objective methods require patients to be observed in a sleep clinic by a sleep expert using costly and obtrusive sensor technology or to only monitor basic sleep measures that are inadequate in order to assess overall sleep quality.

Regarding subjective methods, sleep diaries are one of most common methods for self-assessment. They are easy to use, and they only take some minutes each day to complete. The oldest sleep diary is the Pittsburgh Sleep Diary (PSD) [82], which gathers information that concern factors related to activities during the day (bedtime components) and factors related to last night’s sleep (wake up time components). In addition to subjective methods, sleep questionnaires offer a very cost-effective way to obtain extensive information on sleep patterns, sleep problems, sleep context, and sleep-related behaviors [78]. The Pittsburgh Sleep Quality Index (PSQI) [83], a 19-item questionnaire that assesses sleep quality using subjective ratings for seven different components (i.e., sleep quality; sleep latency; sleep duration; habitual sleep efficiency; sleep disturbance; use of sleeping medication; and, daytime dysfunction) is a widely used questionnaire and scoring tool for sleep quality evaluation. Inside Intelligent Environments, the process of filling-in Sleep Diaries could be simplified by accessing contextual information and inferring the answers a user would provide. If necessary, the system could intervene to ask for a confirmation in order to validate its decisions.

### 2.6. Sleep Improvement Methods

#### 2.6.1. Following Sleep Hygiene Recommendations

Sleep Hygiene [84] provides recommendations for individuals to help them improve sleep quality. In particular, these recommendations are generally aimed at having the individual avoid behavior that interferes with a normal sleep pattern, as well as to engage in behavior that promotes good sleep during the night [85]. Today, there are many different versions of sleep hygiene rules. In some instances, sleep specialists have adopted more limited sleep hygiene rules that only focus on aspects of the sleep environment, effects of exercise, and use of caffeine, alcohol, and nicotine [85]. While taking into account the available versions of sleep hygiene recommendations, below we present the most common sleep hygiene-related factors that influence sleep quality:

- Sleep/Wake up Time Regularity: Sleepers should adhere to regular bed and wake times because this consistency promotes sleep propensity and consolidation [85].
- Caffeine Intake: Individuals should avoid caffeine close to bedtime [86].
- Alcohol Consumption: Individuals should avoid alcohol just before bedtime [85]. Alcohol use 6 h prior to bedtime may lead to significant fragmentation of subsequent sleep, while, alcohol consumption during daytime may lead to sleep latency reduction [86].
- Nicotine Use: Smoking should ideally be avoided, and certainly for at least 2 h before bed. There is little evidence on the effects of nicotine on sleep in nonsmokers [86].
- Nutrition Intake: Food intake timing-mainly in the evening- is negatively correlated with several sleep-related parameters. Moreover, intake of high-fat food is associated with higher sleep latency [87].
- Exercise Regularity: Regular exercise promotes good sleep, but should be avoided close to bedtime [86].
- Stress: Individuals should reduce worry or engage in relaxing activities, particularly before bedtime.
- Noise: Individuals should minimize noise in their sleeping environment [85,86].
- Naps: Daytime napping has been shown to decrease the depth of the major sleep episode and increase latency to sleep onset [85]. Thus, naps are detrimental to subsequent nocturnal sleep.

#### 2.6.2. Creating an Optimal Sleep Environment

Creating good sleep environment conditions (e.g., reduce light exposure, noise) promotes better sleep, as sleep hygiene rules strongly indicate. Scientific sleep studies have shown that poor sleep quality or disrupted sleep could be caused due to environmental factors. However, it is sometimes difficult for a person to assess which factors may be causing disrupted sleep. The parameters that can be manipulated inside an Intelligent space so as to create an optimal sleep environment are listed below:

- Noise: It is easiest to sleep in a quiet place, since sudden or repetitive noises can interrupt sleep. When we hear noise, we may not become fully conscious, but we certainly will come out of the deeper stages of sleep. Interestingly, while certain noises cause interrupted sleep, soft, steady sounds can be soothing [88].
- Temperature: A slightly cool room contributes to good sleep, since a hot environment leads to more wake time and lighter sleep at night, while awakenings multiply. An optimal bedroom temperature is thought to be between 16 °C (60 °F) and 18 °C (65 °F). In most cases, temperatures above 75 °F and below 54 °F will disrupt sleep, but sleep researchers fail to agree on the ideal temperature for sleep. The point at which sleep is interrupted due to temperature or climate conditions varies from person to person and can be affected by bed clothes and bedding materials selected by the sleeper [89].
- Lighting: Lighting affects the perceived atmosphere [67]. Peripheral and non-uniform spatial lighting with low brightness helps to create a relaxing atmosphere, while at the same time, the right color temperature in lighting can be advantageous to human health, well-being, and productivity [90]. Under most circumstances, cool colors have been associated with peaceful, calm, and restful environments, while warm colors are physically and emotionally arousing, exciting, and distracting [68]. In addition, darkness promotes the releasement of melatonin, which relaxes the body and helps to fall asleep faster.
- Electronics and Blue Light: Electronic devices like TVs, computers, tablets, and smart phones, all have a high concentration of blue light. Blue light is the strongest wavelength, and thus the most disruptive to melatonin production. Melatonin is the hormone responsible for sleep which is produced in the evening by the brain. Leaving the lights on and using electronic devices tricks the brain into thinking it is still daytime, thus delaying melatonin production and keeping people awake longer [91]. So, sleep environments should be used only for sleep and any type of electronic device should be avoided just before and during bedtime in order to promote good sleep [89].
- Scents: Another way to make a room feel relaxing is through ambient scent, since specific scents have been shown to have a positive impact on mood [92] and induce relaxation [93]. Aromatherapy, has been used for centuries to promote relaxation, mental and physical wellness. In addition, it is found that it reduces anxiety and increases sleep [94]. In fact, inhaling essential oil molecules may activate brain chemicals involved in controlling sleep [69]. In order to tune the ambient scent, a smart aromatherapy diffuser, which spreads the essentials oils through air without human intervention, can be used.

#### 2.6.3. Following Relaxation Techniques

Relaxation techniques have been a central issue of interest in the areas of medicine, stress management, well-being, and overall lifestyle [95]. Relaxation refers to “a state of relative freedom from both anxiety and skeletal muscle tension” and relaxation response is “a state of decreased psychophysiological arousal: a calming state” [96]. Numerous research studies have extensively examined many different relaxation techniques for several years. In fact, scientifically proven relaxation techniques, in combination with a created relaxing space, can reduce the psychophysiological arousal and improve people’s mental well-being [97]. In particular, many of these relaxation techniques reduce stress and can be further used to help people fall asleep faster in cases where long sleep onset latency is detected. To that end, relaxation techniques have the ability to decrease sleep onset latency and therefore to promote good sleep quality [98]. Most relaxation techniques for sleep include meditation practices, such as diaphragmatic breathing and guided imagery, while others promote music relaxation practices, like listening to relaxing music (e.g., classical music) and sounds (e.g., nature sounds).

## 3. Related Work

### 3.1. Systems Enabling Stress Management

Many studies report their finding on stress recognition or reduction, and there are several applications available, on both iOS and Android, attempting to help users manage their stress. Nevertheless, only a few try to build a complete stress management system, combining both stress monitoring and stress reduction techniques, and even less take advantage of what Ambient Intelligence has to offer.

Hänsel [99] aims to detect the emotions and stress levels of co-workers, using a wearable device and pervasive sensors, and increase mental awareness to improve social connectedness and well-being. For detection, an application for stress self-assessments is deployed on the Apple Watch that measures HR, physical activity, location, and ambient noise. Another application is installed on the phone for additional stress-related questionnaires. Subsequently, a personalized web dashboard presents the collected data to users. The authors also targeted the workplace environment [100], who proposed relating stress at work with daily life events. They used the Discrete Tension Indicator (DTI-2) wristband by Philips Research for continuous sensor measurements, a calendar application, which either stands alone or extracts information from MS Outlook, for collecting information about events at the current time, and a short questionnaire for collecting subjective feedback. In their study, they noticed that even limited information about user’s everyday life makes a difference in achieving stress balance. Bakker et al. [101] had a similar proposal, including additional information from incoming and outgoing mails and messages. Towards helping people cope with occupational stress in the workplace, Trevia [102], in collaboration with Philips Design, designed and built an adaptive relaxation space that was inspired by nature. As a person moves across that space, the environment adjusts its dimensions, light, and sound, relying on his/her position and needs, in order to create a personal or a shared experience of relaxation. Another approach is the one that is described in [103], where the authors propose stress management through a serious game collects information associated with identifying stressors and coping strategies, so as to build the profiles of the players employees. Nevertheless, their proposal is embryonic, and additional information and research is needed.

Serious games for stress management in general are also proposed by [104], where the authors present the concept of Ubiquitous Biofeedback Serious Games (UBSGs) and develop a game for mobile phones, which is called “Botanical Nerves”. Botanical Nerves uses HRV biofeedback to detect stress and a tree as game character. Whenever the user becomes stressed, the tree leaves gradually turn yellow and drop off, and when user relaxes the tree becomes healthy again with more leaves and new flowers. Botanical Nerves seem to effectively reflect and reduce or better control stress. In the same direction, Chen et al. [105] developed a self-paced learning environment, namely FishBuddy, which aims to reduce student anxiety or stress. FishBuddy consists of a self-paced learning system (SPLS), an Apple Watch application, called e-Fish, and a physiologically aware performance evaluation model. The e-Fish application is responsible for detecting stress and anxiety during an exercise, based on student’s heart rate and performance analysis, and reminding the student to watch the fish swimming on the Apple Watch when he/she is stressed.

In the domain of domestic living, Yu et al. [106] designed a pervasive system for stress reduction at home, which uses inter-beat interval (IBI) and HRV to control the lighting in order to provide real time stress response information and help users relax by regulating their breath. Similarly, RESonance [107] follows the same logic, but this time audio biofeedback has also been added, through the natural soundscape, while Inner Flower [108] is a flower-shaped device, which features color LEDs for providing breathing guidance based on HR.

There are also many stress relief apps in the global market, which some people might find very useful, but they do not make use of Ambient Intelligence technology. Calm (https://www.calm.com/ accessed on 3 February 2021) is one of the most popular, which offers guided meditations, sleep stories, music tracks, video lessons on mindful movement and gentle stretching, nature scenes and sounds, and audio programs, and its aim is to reduce stress, anxiety, and improve sleep quality. Moreover, there are some mobile apps that use biofeedback devices for stress management, such as Pip (https://thepip.com/en-eu/ accessed on 3 February 2021), which is a small wireless device that monitors EDA signals and works together with mobile apps that use audiovisual feedback to present users’ stress levels.

Detecting stress levels and making attempts toward reducing it has been a topic of growing research interest, as well as commercial interest, as analyzed above. Most existing systems are able to identify a user’s stress levels, but they refrain from utilizing ambient intelligence facilities in order to make stress detection more accurate. For example, when the user is excited, because his/her favorite TV show is just beginning, the identification of a peak in physiological signals may lead to misleading results (i.e., detecting high stress levels), if the system does not take contextual information into consideration. In addition, most of the existing approaches include mechanisms to reduce stress when identified, but they rely on a single device application or require the user to be in a specific setting.

### 3.2. Systems Improving Sleep Quality

There are several research studies that have investigated numerous sleep technologies, as well as approaches to monitor sleep behavior at home by collecting information regarding individuals’ sleep patterns. Most of them are mobile applications that utilize the built-in phone sensors to monitor sleep behavior and predict its quality. For example, the BES model [109] uses a sensor-based inference algorithm to predict sleep metrics by exploiting various usage patterns (e.g., screen time) and environmental observations (e.g., prolonged silence). In addition, iSleep [110] and Toss ‘N’ Turn [111] capture data from phone sensors (e.g., accelerometer) to detect sleep measures (e.g., duration, disturbances) and infer sleep quality by using a daily sleep diary based on the PSQI. Other approaches track sleep patterns in an unobtrusive way, such as DoppleSleep [112], which tracks an individual’s physical body movements, heart beat, and breathing during sleep by using a single Doppler radar sensor to objectively infer sleep quality. Finally, efforts have been made to promote sleep hygiene in order to help individuals improve their sleep quality. For example, SleepTight [113] provides feedback to help people change their behavior by capturing sleep measures from a sleep diary and contributing factors (e.g., exercise, caffeine intake). Similarly, ShutEye [114] uses the phone’s wallpaper to inform users about how their activities may disrupt their sleep.

Although many of the approaches are still running in sleep labs, in recent years consumer sleep technologies have appeared on the market. They include mobile device applications (utilizing mobile device functions such as the camera or microphone), wearable devices, embedded/contactless devices (integrated into furniture or other artifacts in the sleep environment), and accessory appliances (like smart lights and alarms). Regarding applications for mobile devices, ‘Sleep Cycle’ (https://www.sleepcycle.com/ accessed on 3 February 2021) and ‘Sleep As Android’ (https://sleep.urbandroid.org/ accessed on 3 February 2021) are two well-known commercial applications that utilize the mobile device’s built-in sensors to collect sleep-related data, including sleep metrics (e.g., sleep duration) and bio-signals (e.g., heart rate). Moreover, they provide a smart alarm feature to wake up users during their lightest sleep phase as well as sleep recommendations in order to encourage users to adopt better sleep routines. In addition, many of these applications aim to relax users in order to help them fall asleep faster and effortlessly by exploiting appropriate relaxation techniques, such as meditation (https://www.calm.com/ accessed on 3 February 2021), storytelling (https://www.headspace.com/ accessed on 3 February 2021), and relaxing sounds, such as forest soundscapes (https://www.relaxmelodies.com/ accessed on 3 February 2021).

Recently, plenty of wearable devices have appeared on the market, mainly for the user’s wrist (e.g., activity trackers, such as Withings STEEL HR (https://www.withings.com/ca/en/steel-hr accessed on 3 February 2021)) or head (e.g., headbands, such as Dreem 2 (https://dreem.com/en/ accessed on 3 February 2021)). These devices collect sleep-related parameters (e.g., sleep stages), bio-signals (e.g., brainwave activity), as well as activity metrics (e.g., workouts) in order to estimate the overall sleep quality. Furthermore, significant efforts have been made to create sleep trackers that are placed under the mattress and monitor sleep patterns in an unobtrusive and contactless way (e.g., Withings Sleep Tracking Mat (https://www.withings.com/mx/en/sleep accessed on 3 February 2021)).

## 4. The Intelligent Home of ICS-FORTH

### 4.1. Intelligent Spaces

The Intelligent Home of FORTH-ICS is a two-story apartment that is located in the AmI Research Facility of the Institute. Inside this environment, everyday user activities are enhanced with the use of innovative interaction techniques, artificial intelligence, ambient applications, sophisticated middleware, monitoring and decision-making mechanisms, and distributed micro-services. Regarding the programmable hardware facilities of the Intelligent Home, they currently include commercial devices (e.g., smart lights, motorized blinds, smart speakers, smart oil diffusers), appliances (fridge, oven, coffee machine, air-conditioner), and technologically augmented custom-made artifacts. From an engineering perspective, the Intelligent Home’s software infrastructure is built around the microservices architecture [115]. Every component (e.g., physical artifact, digital object, smart service) exposes its functionality as a service to a collection of advanced frameworks and tools that: (i) enable residents to personalize the behavior of their surroundings in a user-friendly manner [116] and (ii) the intelligent environment to seamlessly orchestrate the operation of heterogenous systems [117]. Even the various User Interfaces are implemented using a MEAN stack architecture [118], and they expose a full-blown API to the environment (and co-located third-party components) so as to enable their external manipulation (e.g., move to a specific screen, initiate an activity with certain parameters).

**Figure 1:**
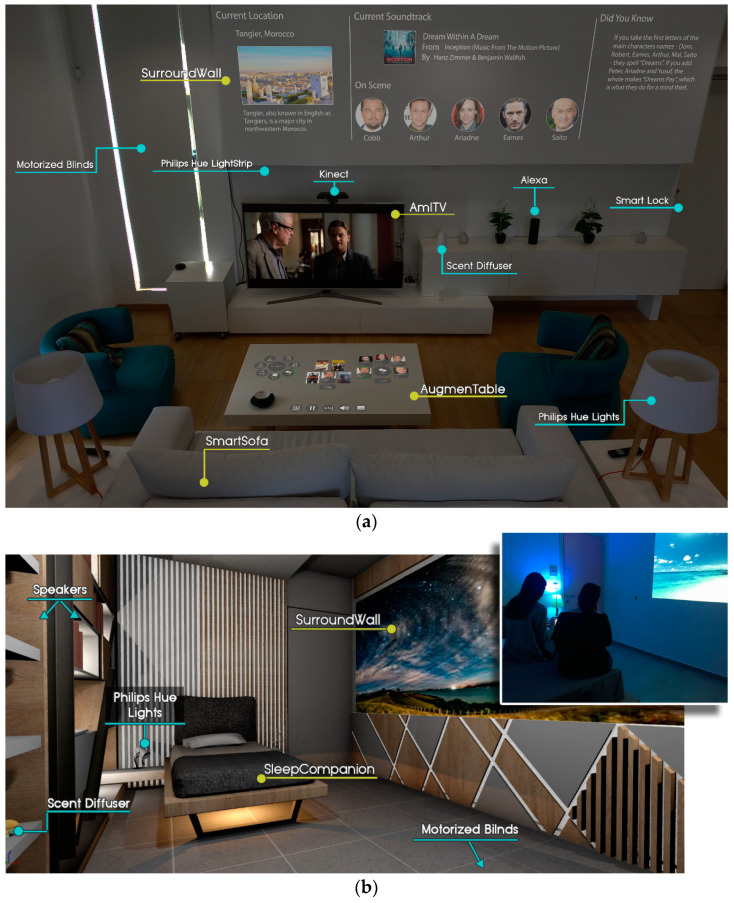
(**a**) the Intelligent Living Room; and (**b**) the Intelligent Bedroom.

The living room of the Intelligent Home [119] is equipped with three (3) technologically augmented artifacts, namely AugmenTable, SurroundWall, and SmartSofa (Figure 1a). AugmenTable is a commercial coffee table that is made of wood with a smooth, non-reflective white finish that acts as a large projection area, while, through a Microsoft Kinect sensor, it becomes a touch-enabled surface that can recognize the physical objects placed on it. Additionally, SurroundWall transforms the wall around the TV into a secondary non-interactive display that provides an enhanced viewing experience by augmenting—in a context-sensitive manner—The content presented on the TV. Finally, SmartSofa is a commercial sofa that is equipped with various sensors aiming to detect user presence inside the room and provide information regarding the user’s posture while seated.

Regarding the bedroom of the Intelligent Home (Figure 1b), it currently features a technologically augmented single bed, named SleepCompanion, integrating various sensors like Withings Sleep Tracking Mat (https://www.withings.com/mx/en/sleep accessed on 3 February 2021), and Emfit QS Sleep Tracker (https://www.emfit.com/ accessed on 3 February 2021). Additionally, a variant of the SurroundWall artifact is also installed inside this room, which projects on a wall parallel to the bed.

### 4.2. AmIHomeOS

The Intelligent Home of ICS-FORTH is equipped with a sophisticated mechanism, namely AmIHomeOS, which aims to transform the house into an all-inclusive environment that assists users in a personalized manner throughout their daily activities. AmIHomeOS is based on a collection of programs, called AmI Scripts [117], which are able to (i) control any programmable artefact, (ii) exploit contextual information to make “informed” decisions, (iii) personalize the delivered content according to users’ preferences, and (iv) expose the functionality of the intelligent artefacts as a service in order to enable integration with third-party tools. In more detail, it is a collection of distributed, isolated microservices that operate autonomously, but also expose their functionality and data to be used as part of the wider ecosystem of the Intelligent Home. In order to ensure privacy, any data shared across the various home services are processed locally and remain within the home’s sandboxed environment. Τhe currently available services are described below:

- User preferences and profile: provides access to users’ personal data, characteristics and configuration parameters.
- User tracking: monitors the presence of people and tracks their movements in the surrounding environment.
- User activity tracking: keeps track of the activities that a user is engaged with; for every activity the completed, ongoing and future steps are available (e.g., step 7 out of 15 in preparing dinner).
- User health state: this service stores—in a timely manner—various health-related information and exposes both aggregated and detailed (recent) data.
- User stress levels: provides access to users’ current and past stress levels, along with the identified stress factors.
- User sleep data: provides access to various sleep-related information (e.g., sleep duration, sleep score, time to fall asleep, snoring activity).
- User agenda: allows an ambient application to access and modify the user’s appointments, tasks, meetings, and events.
- Nutrition- and Diet-related services: such services provide information regarding the nutrition and ingredients of recipes, but they can also provide details regarding the meals that an individual had (e.g., type of meal, time that was consumed, quantity).
- Relaxation Programs: this service defines how the Intelligent Environment should be adapted in order to satisfy the requirements of a relaxation technique. It provides access to content (e.g., specific multimedia files) and information regarding the available/appropriate devices (along with their attributes) of a specific environment.
- Home Control: holds the current state and permits control of every application/device/service that is part of the Intelligent Environment (e.g., bed-side lamps are on, cake should be baked for seven more minutes).

## 5. Design Process

An iterative, User-Centered Design methodology [120] was followed for the design of CaLmi and HypnOS, with special attention being given to the pre-design stages, as described in the Design Thinking process [121]. “Design Thinking is a non-linear, iterative process which seeks to understand users, challenge assumptions, redefine problems and create innovative solutions to prototype and test” [122]. This method consists of five phases: Empathize, Define, Ideate, Prototype, and Test.

The Empathize step of the process requires gaining an empathic understanding of the problem under investigation. To that end, HypnOS’s design team (i.e., designers, interaction designers, and UX experts) consulted with sleep experts from a sleep research laboratory, (Sleep Disorders Center, Department of Respiratory Medicine, Medical School, University of Crete, Heraklion, Crete) in order to gain valuable feedback and insights. As an outcome, it was suggested that HypnOS should not provide medical-level services, since the required equipment for retrieving medically acceptable data would make the system far too obtrusive for a home environment. Additionally, it was made clear that patients in sleep labs are often overwhelmed by the level of surveillance, which includes electrodes that are attached to various places on the body and head, as well as cameras recording the patient all night, which results in discomfort and it is generally an unpleasant experience. Therefore, in the Define step of the process, it was decided that the scope of the system would be to monitor, as unobtrusively as possible, the sleep behavior of the individuals in order to identify their sleep patterns. It was also suggested that, in combination with the monitoring of their daily habits—through other ambient monitoring services of the Intelligent Home—the system could offer valid insights in form of advice to improve sleep hygiene and, therefore, their sleep quality.

Similarly, the design team of CaLmi (i.e., designers, interaction designers, and UX experts) consulted with one occupational therapist and one expert in relaxation techniques during the Empathize and Define steps of the process in order to gain the best possible understanding of when and how the system could assist users. During these steps, the design team gained a good understanding of potential end-users and relaxation methods. Additionally, it was pointed out that each individual user has different needs, and it is not possible to achieve stress relief by offering them the exact same experience. Therefore, it was made clear that the relaxation programs should be highly customizable and adaptable to the user’s needs and preferences, in order to achieve a state of relaxation. Moreover, it was suggested that, in addition to the physiological signals, the monitoring of the user’s daily habits should be exploited to improve stress monitoring and offer valid insights.

Next, in the Ideation process of both systems, several brainstorming sessions took place, during which dozens of ideas were produced and then filtered appropriately. By properly shaping those ideas, the preliminary requirements for both HypnOS and CaLmi were extracted. In the Prototype phase, the design team of each system followed an iterative design process, where low and high-fidelity interactive prototypes were created for the end-user applications. Moreover, before proceeding with user testing, the overall setup and the application prototypes were iteratively evaluated by experts so as to uncover issues in terms of interaction and ergonomics.

## 6. The CaLmi System

### 6.1. Overview

The vision of CaLmi is to create a pervasive system for Intelligent Homes that reduces the stress of their inhabitants [97]. Particularly, the system aims to detect, as unobtrusively as possible, whenever a user is stressed and try to help him/her relax by exploiting a variety of devices. The end-users will be able to monitor their stress levels in real time and accept automatic personalized relaxation suggestions from the system, or to voluntarily activate any of the system’s relaxation programs. In the long run, the system aims to help users live a less stressful life and improve their mental well-being.

A wireless wearable device, as well as the technological equipment and installations of the Intelligent Home are used for the detection of the stress state. In more details, CaLmi employs a wristband that collects user biometrics, while it utilizes various contextual data in order to better understand the user’s daily activities, health, nutrition, and sleep quality.

**Figure 2:**
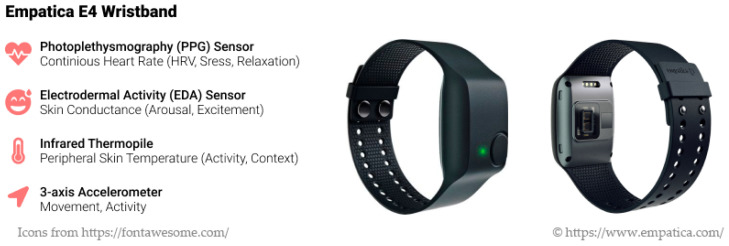
Empatica E4 wristband’s sensors (https://www.empatica.com/en-eu/research/e4/) (accessed on 3 February 2021).

An Empatica E4 wristband is the specific wearable device that has been selected for collecting physiological signals for stress monitoring. E4 is designed specifically for research that is conducted both inside and outside the lab. CaLmi uses the E4′s PPG sensor to obtain blood volume pulse (BVP), from which the HRV can be derived, the EDA sensor to obtain EDA data, and the infrared thermopile to derive peripheral body temperature (Figure 2). Moreover, it uses the accelerometer, which captures motion, to disambiguate whether changes in physiological signals are caused by stress or physical activity [123]. In daily life, users need to wear the E4 on their wrist to allow system access to their physiological signals. Accordingly, the system continuously monitors these signals and, when a peak is identified, it uses contextual information to determine whether a stressful situation, which should trigger an intervention, has occurred.

CaLmi introduces custom context-sensitive micro-reasoners that are used to identify stressful situations or behaviors that indicate the user is stressed. The identification process is based on the analysis of data about the user’s daily activities, habits, responsibilities, duties, and appointments that can be retrieved from the ambient facilities of the Intelligent Home (Section 4.2). For example, a stress indication is detected if a significant increase in calories is recorded by the Nutrition service and the user tends to overeat when he/she is stressed.

Upon stress detection, CaLmi offers different relaxation programs in order to reduce stress, accommodating multiple users. It must be taken into consideration that something relaxing for one person can be annoying for another. Therefore, the right choice and use of a program is crucial. Additionally, the stress management application distributes across multiple devices and cooperates with other smart facilities (e.g., ambient lighting, notifications system, entertainment system) according to user preferences, so as to create a relaxing environment at home.

Before executing any relaxation program, the user is prompted to get in a comfortable position. Additionally, except from the case where natural light is necessary for reading and/or writing, the motorized blinds of the room are closed. When the system identifies that the user is ready to begin, the program is launched. Based on the stress reduction methods that are discussed in Section 2.3, the available relaxation programs are:

- Exposure to Nature: the main objective of this program is to make users feel like they are outdoors in a natural environment, such as a forest. In order to achieve this, when inside the intelligent home, the system can utilize all of the available artifacts of the user’s current room location. A collection of slideshows or videos is displayed in the largest and most convenient display area, while the lights adapt to a natural color, according to the displayed pictures or videos (e.g., when a sunset by the sea video is shown, lights take an orange hue). In addition, corresponding sounds can be heard from the sound system and the room is filled with a natural essence (e.g., when simulating a forest, the room takes a cypress scent).
- Meditation: this program gives the user the opportunity to practice a variety of meditation techniques, such as body scan, sitting meditation, and mindful hatha yoga. It consists of audio instructions and, when needed, guided meditation videos. Some of the techniques may require the user to have closed eyes, in which case meditation instructions are only given through the sound system, otherwise an instructional video is displayed on the screen closest to the user. In addition, the intelligent home automatically lowers the lights and a relaxing scent (e.g., sandalwood) fills the room.
- Relaxing Music: the sound system takes center role in this program. Relaxing songs (e.g., classical music tracks) can be heard from the room’s speakers and the lamps generate lighting patterns in real time based on the sound. Moreover, the user may choose to additionally use the aromatherapy system.
- Expressive Writing: this program consists of textual forms with questions that help the user to express their thoughts and feelings about problems of his/her everyday life that have affected their stress levels. The system supports multimodality by enabling the user to either express himself/herself in writing form or in a more natural way using his/her voice, which is then automatically transformed to text. While the user is interacting with the system, his/her written thoughts are displayed on the most convenient display. Additionally, the lighting in the room should be at appropriate levels for reading and writing. The user can also use the aromatherapy system in the same manner as the previous program.
- Customizable Programs: the developed relaxation player can be employed to support any of the aforementioned programs, either individually or in combination. For example, the user could be listening to music while he/she is writing about his/her feelings. This is possible, since the user is able to create his/her own relaxation program by combining the existing components. For example, the user could choose to display old family photographs on TV and his/her current stress level chart on the coffee table (which are two of the available display areas in the room he/she is currently in), to play Bach’s Prelude from the room’s speakers, not to use the scent diffuser, and to reduce the brightness of the lights.

**Figure 3:**
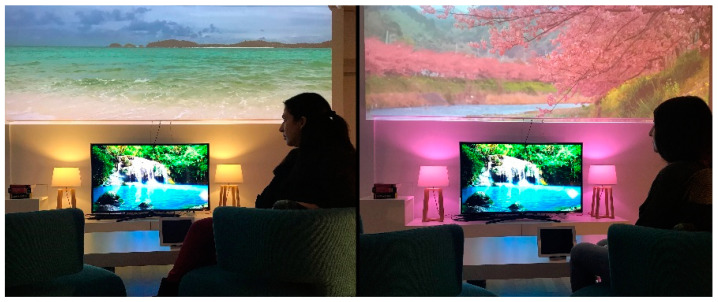
Relaxation technique “Exposure to Nature”, adjusted to user preferences.

The most appropriate relaxation program is selected whenever high stress levels are detected or the user just wants to relax. Measuring and comparing user’s stress levels before and after each program execution allows for observing their effectiveness and make appropriate decisions. The available programs can be customized according to the user’s preferences. For example, some people enjoy better videos of natural landscapes rather than a beach view (Figure 3). To this end, when employing the technique “exposure to nature”, the system has the ability to adapt according to the characteristics of the individual user. In addition, the program is selected according to the current user needs and context of use (e.g., user’s current state, time constraints). For example, if a user is lying in bed, but cannot sleep because of stress, even though it has been observed that “Expressive Writing” is the most effective program for him/her, “Relaxing Music” (the second-best option in terms of effectiveness) is selected, since it does not require mental effort or bright lighting, which might disrupt sleep. Furthermore, the user can optionally configure or (de-) activate various features of the predefined programs.

Nevertheless, the choice of the relaxation technique is not the only parameter that should be taken into consideration. The appropriate hosts (e.g., devices, artifacts) for the selected program must be also selected. Thanks to the ambient facilities of the Intelligent Home, CaLmi has access to information regarding the user’s current position and it can easily select the artifacts that are closer to him/her (e.g., TV, SurroundWall, odor diffuser, speakers). Correspondingly, if the user is in a room with other individuals, the system will suggest moving into a different one, so as to ensure that the relaxation program will be effective. Additionally, in case the user is not at home, he/she can use their mobile device to experience the selected program via the available modalities (i.e., touch display and sound output).

Moreover, CaLmi employs a reward mechanism (i.e., gamification) that aims to motivate the user in following the suggested relaxation methods and, thus, learn to relieve tension and live a less stressful life. The mechanism works, as follows: certain tasks, which have various levels of difficulty, are assigned to users, who have to fulfill these tasks in order to earn trophies. A trophy corresponds to the completion of one task. Some of the tasks are: have your first relaxation session, try every relaxation program, have sessions several days (i.e., five, 10, or 20) in a row, complete a specific number of relaxation sessions (i.e., 10, 20, or 30), and achieve stress reduction during a particular period of time (i.e., one week, month, or year).

### 6.2. Architecture

**Figure 4:**
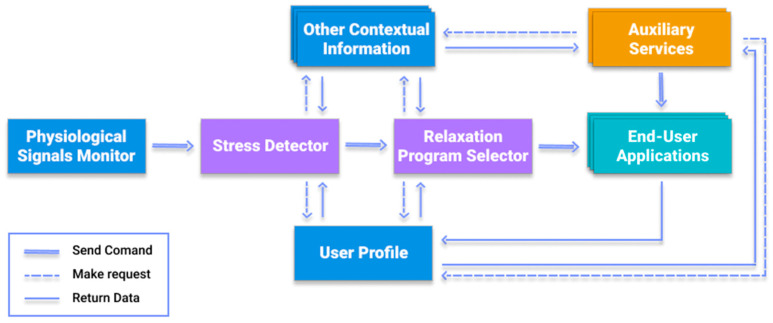
High level architecture of CaLmi.

Figure 4 illustrates an of the CaLmi’s architecture. As a first step, CaLmi continuously monitors user’s physiological signals and then informs the stress detector when peaks are identified. After receiving a peak detection notice, the stress detector requests the user profile and current context information to determine whether or not it is caused by stress. If stress is the cause of the peaks, the relaxation program selector is informed in order to select a relaxation program that fits user’s preferences and the current context. Once the program has been selected, the user is advised to start it and interaction with the system begins. Upon completion of the program, the end-user applications return valuable data to the user profile in order to update it. Additionally, the auxiliary services periodically request the user profile and contextual information to detect situations where users need stress-related advice.

CaLmi employs the Empatica E4 wristband to obtain user’s physiological signals and make a first estimate of his/her stress level. The E4 streaming server connects to the E4 wristband and forwards its data streams to the E4 client, which stores them in the InfluxDB database. Then the E4 client and EDA explorer [124] retrieve data from the InfluxDB in order to calculate the user’s stress percentages and search for peaks.

The stress detector starts the process of confirming that the user is stressed when it receives an HTTP request from the EDA explorer. In more details, it requests the user profile and current context information from the custom context-sensitive micro-reasoners to determine whether the peak that is detected in the physiological signals from the EDA explorer is caused by stress. Therefore, the question that must be answered (with 1 or 0, where 1 = yes and 0 = no) by the stress detector based on the data obtained from the current context and the user profile is: “Is the user under enough stress to require a remedial action?” The stress detector uses the binary logistic regression algorithm to answer that question. Logistic regression [125] is a commonly used machine learning algorithm for predicting the probability of an event occurring based on previous given data. The probability, which has been calculated by the algorithm, must be greater than 75%, in order for the user to be classified as highly stressed. In that case, the stress detector sends an HTTP request to the relaxation program selector in order to select the appropriate intervention. Otherwise, the user is not classified as highly stressed and there is no need to select a relaxation program.

The relaxation program selector starts the process of choosing the relaxation technique when it receives an HTTP request from the stress detector. Time constraints and the desired activity level play a critical role in that decision. The data obtained from the smart calendar (e.g., a nine o’clock appointment) and processed by a micro-reasoner (e.g., user has ten minutes available) determine the maximum duration of the session. In addition, the data obtained from the user activity tracking (e.g., user is lying in bed) and processed by a micro-reasoner (e.g., low activity level is desired) determines the desired activity level. Thus, the relaxation program selector chooses the most efficient program for the user based on his/her profile that satisfies the timing and activity level constraints and customizes it according to his/her profile preferences. However, the process stops if there is no time for starting a session. After making the program selection, the hosts must be chosen. The data obtained from the user activity tracking determine the user’s current room location and if there are other people in the same room. In case he/she is alone in the room, the appropriate hosts (e.g., TV, speakers, Wall Projector), which are nearest to him/her, are selected. Otherwise, the appropriate hosts of the nearest available room are selected, and it is suggested to the user to move into that room.

The end-user applications are associated with any procedure responsible for the intercommunication between the system and user. Finally, a collection of auxiliary services provides users with tips for precautionary purposes and advice when suspicious stress patterns occur. The schedule tracker and the chronic stress detector detect situations where users need stress-related advice and display the appropriate messages to them through the end-user applications.

### 6.3. End-User Applications

With the intention of communicating valuable information to the user, CaLmi exploits the pervasive notification system of the Intelligent Home. Specifically, the nearest intelligent device can display messages, including: (i) guidelines to assist stressed-out users, (ii) information on stress levels after the completion of a relaxation program, (iii) notifications after winning a trophy, (iv) advice to plan pending tasks in advance, when user’s schedule seems overloaded, and (v) guidance regarding requesting professional help the in case of consistently high stress levels.

Moreover, a pervasive relaxation player application has been developed to assist users by providing appropriate interventions, which are presented in the form of relaxation programs. Each program is created by defining specific attributes. In more detail, exploiting the ambient facilities of the Intelligent Home, the player has the ability to project multimedia to a room’s display areas (i.e., surroundWall, AugmenTable, and TV) (Figure 1a), play sound and music from the room’s speakers, adjust a room’s lighting conditions (via Philips Hue Lights and Lightstrip), release a pleasant odor into the room using a scent diffuser, etc.

**Figure 5:**
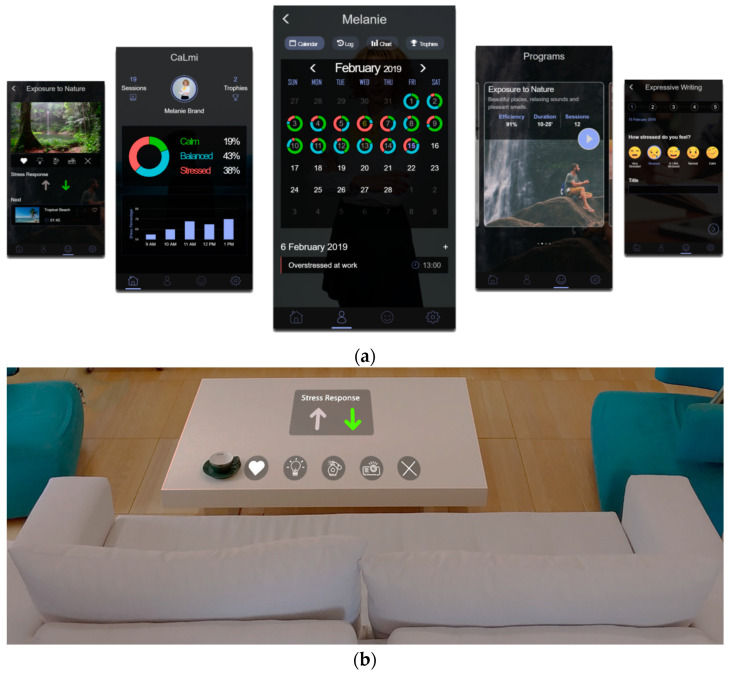
(**a**) User Interface of CaLmi’s mobile application; (**b**) Real time stress response and session’s control menu on the coffee table.

A user-friendly mobile application has also been developed to facilitate control over the system and assist the user (Figure 5a). By using his/her tablet or smartphone, a user can access the application from everywhere; in more details, the user can inspect his/her own or other family members’—respecting their privacy settings—stress levels and patterns over a specific period of time. Furthermore, a user can access a history log describing the stress levels at each time period, and present information regarding the efficiency of the selected relaxation techniques (stress levels before and after, time, date, location, events and people nearby, and user’s feedback). The purpose is to make the user understand the roots of stress and the techniques that calms him/her down. Additionally, the application is able to display the user’s achievements and trophies that have been collected through systematic use. Finally, there is the ability to manually explore and try various relaxation programs and techniques.

Additionally, CaLmi offers interfaces (UIs) that can be hosted by the various technologically enhanced artifacts of the Intelligent Home (Section 4). For each of them, it considers the existence and dimensions of the display, the available input controls, and various other aspects that affect interaction, so as to deliver user-friendly and usable interfaces. For example, during a relaxation session, which takes place in the living room, users are able to view the real time visualization of their stress fluctuations and the session’s control menu on their smartphone (Figure 5a) or on the AugmenTable (Figure 5b).

### 6.4. Evaluation

A formative evaluation of the CaLmi system was conducted with the participation of eight users, three females and five males, in the living room of the Intelligent Home of FORTH-ICS. The age of the participants varied from 25 to 54 years old. People who were allergic to the selected aromatherapy essence (i.e., lavender) and to the materials of the Empatica E4 wristband (i.e., plastic, silicone, silver, gold, and brass) were excluded from the evaluation. Furthermore, people who had been given medication that could affect the physiological signals (e.g., anxiolytics, antidepressants, diuretics, and blood pressure, thyroid, diabetes, or heart disease medications), had been suffering from substance abuse that could affect the physiological signals (e.g., alcohol, drugs), or traveled across more than one time zone the week before the evaluation [126,127,128] were also excluded in order to ensure that the signals were reliable without having to personalize the system for each participant.

In the context of the evaluation process, the European Union (EU) regulation on General Data Protection (GTPR; 2016/679) has been properly taken into account and approval has been obtained by the FORTH Ethics Committee. In addition, the participants were given information about the nature of the evaluation and all aspects of participation, and a consent form was signed. The evaluation process consisted of three phases:

Phase 1—Initial Data Collection. For two consecutive days, the participants were requested to wear the Empatica E4 wristband for as long as possible in their daily lives. During that time, they were also requested to write down, in two printed forms (one for each day), the times of the day they thought that there were increases in their physiological signals, due to (a) a stressful event, (b) physical exercise, or (c) some other cause without specification. The above information was later (a-posteriori) analyzed to demonstrate each participant’s physiological signals range and identify the values that are likely to signal high stress levels.

Phase 2—Relaxation Program Testing. In the following three days, the participants were requested to continue wearing the Empatica E4 wristband, and they were asked to follow the same relaxation program via (i) CaLmi (multisensory session) and (b) a tablet device (monosensory session), sometime that they felt stressed. The participants had the flexibility to select which session to attend first, while both sessions should take place in the living room of the Intelligent Home. The relaxation program available to the participants was the “Exposure to Nature”, and the two sessions were structured, as follows:

- Multisensory session: (a) displays a video of a forest waterfall on the main living room wall and adjusts the color (i.e., it takes a greenish-blue hue of the waterfall’s water) and the intensity (i.e., it decreases) of the room lighting, to activate the sense of sight. (b) Reproduces relaxing music and forest sounds (e.g., running water and birdsong) from the room’s speakers, to activate the sense of hearing. (c) Releases the lavender scent using the scent diffuser to activate the sense of smell.
- Monosensory session: displays the same video of a forest waterfall on a soundless tablet device to activate only the sense of sight.

**Figure 6:**
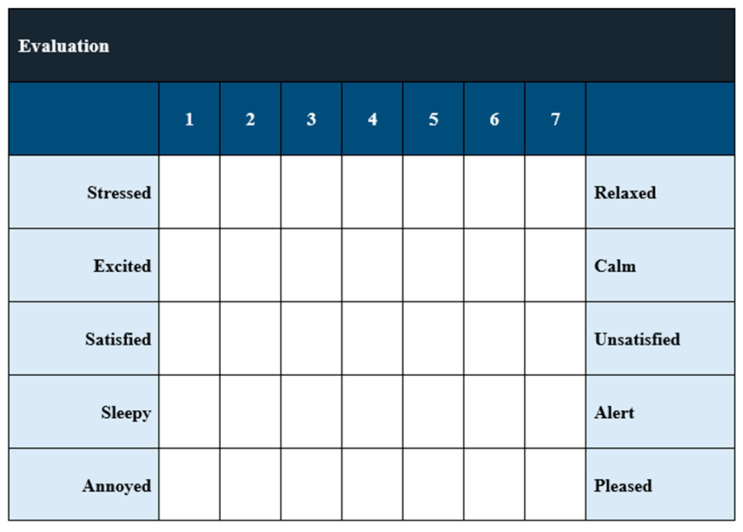
Post-session questionnaire for the evaluation of CaLmi.

The participants continued to wear the Empatica E4 wristband during the sessions, and one hour after their completion, in order to record their physiological signals and, thus, possible changes in stress levels. Moreover, after each session, the participants were asked to fill the questionnaire that is presented in Figure 6.

Phase 3—Debriefing. After the completion of Phases 1 and 2, interviews regarding participants’ experience with the system were conducted in order to report their comments and general opinion.

**Figure 7:**
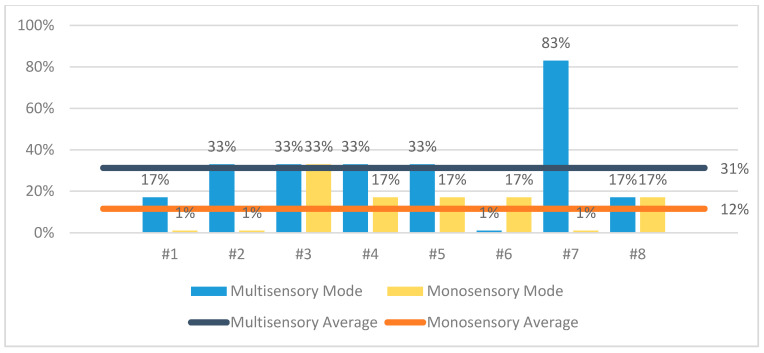
Subjective stress reduction per participant οf the CaLmi evaluation.

**Figure 8:**
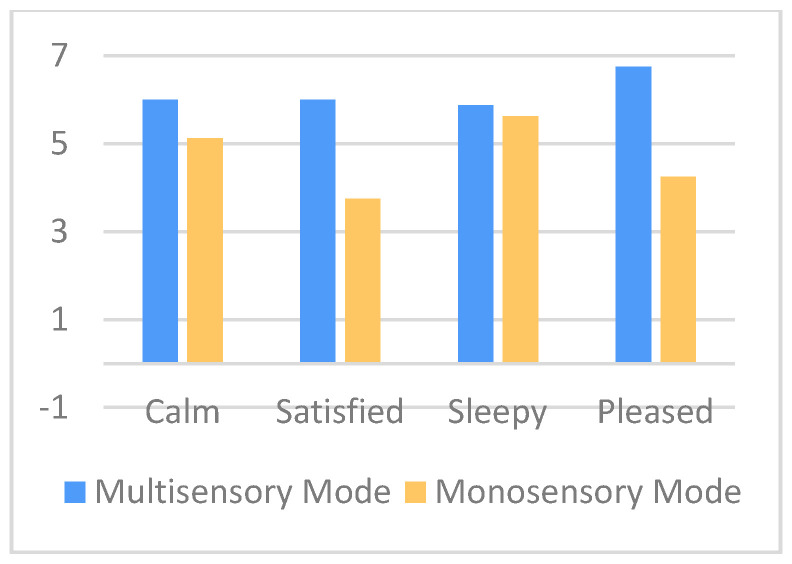
How participants felt after each session οf the CaLmi evaluation.

**Figure 9:**
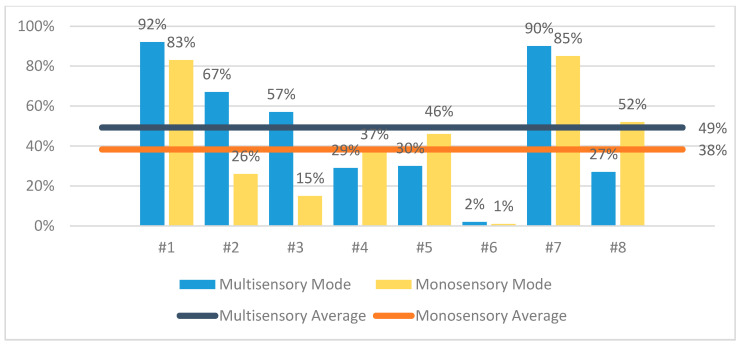
Objective stress reduction per participant οf the CaLmi evaluation.

The results of the evaluation confirmed that CaLmi offers a more effective and satisfying relaxation session by using the technological equipment and installations of the Intelligent Home to activate different senses, rather than a single device that is limited to the visual sense alone (monosensory mode). According to the information that was provided in the questionnaires, 62% of the participants thought that they were less stressed after the multisensory session in comparison with the monosensory (Figure 7), while all of the participants felt calmer, satisfied, sleepy and pleased after using CaLmi (Figure 8). This finding is corroborated by the EDA signals, which revealed that 62% of the participants were calmer after the multisensory session in comparison to the monosensory (Figure 9). In more detail, all participants, except one, seemed to be more relaxed after the relaxation sessions and their EDA values were reduced by at least 29% and, on average, by 49% after the multisensory session, while, in the best case, it reached 92%. Regarding the participant whose EDA values were not reduced after the multisensory session (remained the same), it was observed that the mososensory session was not effective either (EDA values increased). We hypothesize that the reason for this outcome is the fact that he/she had drank more than three coffees before both sessions, which may have affected his/her experience. Some of the participants found the sound more relaxing and others the lighting and the big projection area, while all enjoyed the combination of sight, sound and scent. One of them stated that it was the perfect combination (neither incomplete, nor overloaded) and another one that he/she felt like being there, in nature. Moreover, the participants stated that they would use CaLmi in their everyday lives in order to receive multi-sensory, context-aware, personalized help for stress reduction.

## 7. The HypnOS System

### 7.1. Overview

**Figure 10:**
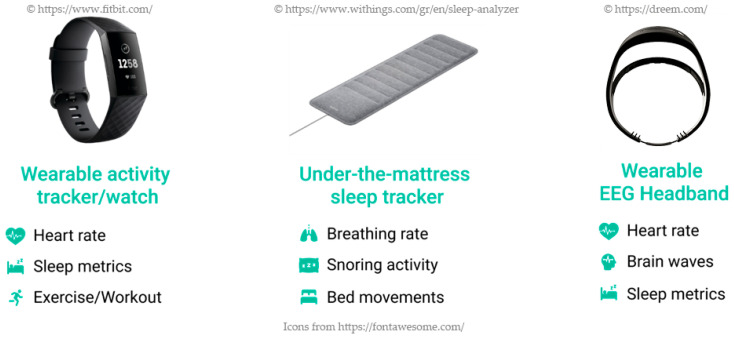
Sleep trackers utilized by HypnOS.

The HypnOS system is an unobtrusive sleep monitoring and recommendation system for Intelligent Homes. It is able to track sleep activity, detect sleep abnormalities, and assess sleep quality by monitoring resident’s sleep-related parameters (e.g., sleep duration, snoring activity), bio-signals (e.g., heart rate, breathing rate), daily habits (e.g., caffeine and alcohol consumption), subjective measurements (e.g., sleepiness feeling, subjective sleep quality), and contextual information (e.g., daily activities, nutrition). For the collection of the required information, it utilizes sleep trackers (Figure 10), the ambient monitoring services of the Intelligent Home (e.g., nutrition micro-service), and a daily sleep diary that is offered by the HypnOS’s mobile application.

HypnOS employs a group of wireless sleep trackers, including a wearable activity tracker/watch (e.g., Fitbit Charge 3, Withings STEEL HR), an under-the-mattress sleep tracker (Emfit QS, Withings Sleep), and a wearable EEG headband (Dreem 2, BrainBit). These devices are equipped with a large number of sensors, such as 3-axis-accelerometer, which tracks motion patterns, a pulse oximeter, which tracks heart-rate, a pneumatic sensor for breathing rate tracking, a ballistocardiography sensor for continuous heart and breathing rate, EEG sensors for EEG brainwaves monitoring, and a microphone for snoring detection. These devices monitor sleep and bio-signal data and, when new data are available, they are propagated to the sophisticated decision-making mechanisms of HypnOS.

Apart from relying merely on sleep measurements and bio-signals, HypnOS takes the context of use into account so as to gain insights about the causes of the detected sleep-related issues and act accordingly. In order to do so, it utilizes many of the services described in Section 4.2. In particular, the nutrition service provides all of the information regarding the inhabitants’ daily food consumption (e.g., type of meal, calories), which is very important considering that eating habits can also affect sleep. More specifically, eating too close to bedtime may disrupt sleep during the night. Moreover, certain foods (e.g., diets high in fat and junk foods) will have negative effects on sleep quality, while foods that are rich in tryptophan (e.g., milk, cheese, crackers)—an amino acid that causes sleepiness—may lead to better sleep quality. Furthermore, it is important for HypnOS to be informed regarding the residents’ activities throughout the day. To this end, it consumes information from the user activity tracking service. Being awakened or out of bed (e.g., use of the bathroom several times), being irritated or uncomfortable due to others (e.g., bedpartner, pets or children), doing arousing and stressful activities (e.g., working, watching TV) before bedtime are conditions that the system should be aware of, so as to identify abnormal or unexpected user behaviors. Additionally, it utilizes the stress service so as to understand whether a user has high levels of stress. Stress can impact daily life in many ways, including negatively affecting the quality of sleep; a very common phenomenon is when people lying in bed worry and feel anxious, which makes it almost impossible to relax and quiet their mind enough to fall asleep [129]. In fact, all types of stress can harm sleep quality and sleep deprivation can fuel further stress and irritability. Finally, with the help of the home control service, HypnOS can identify, in real time, the environmental conditions of the bedroom, and adjust them according to the optimal conditions for a restful sleep (e.g., temperature for best sleep is between 60 and 67 °F, bedroom humidity level to 40%, and light brightness to 30%). This is of outmost importance when considering that poor sleep quality or disrupted sleep could be caused due to environmental factors in the sleep environment. Ideally, the sleep environment should be a sanctuary for restful and restorative sleep. Environments that are noisy, bright, warm, full of moisture, and bad air quality affect negatively the sleep and wake up process. Unfortunately, it is sometimes difficult for a person to assess which factors in the environment may be causing disrupted sleep, which highlights the necessity for a system like HypnOS.

HypnOS also relies on a sleep diary in order to assess the sleep quality of a user. In sleep medicine, the sleep diary is considered to be the “gold standard” for subjective sleep assessment [130]. In general, sleep diaries allow people to self-assess their sleep by collecting data every day. It is usually completed over a period of time (usually one or two weeks). This means that sleep diaries collect lots of sleep-related information, and also that this kind of information is precise enough. Moreover, it is not so dependent on memory, because it is often filled-in just after waking up and/or just before going to sleep. There are also mobile applications that act as electronic sleep diaries, which are similar to the paper-based ones with respect to their diagnostic power [131].

Today, there are several sleep diaries available. In our endeavor to create a custom sleep diary as a supplementary functionality to the basic monitoring, the most popular sleep diaries (e.g., Pittsburgh Sleep Diary (PSD) [82]), Consensus Sleep Diary (CSD) [130], and National Sleep Foundation (NSF) [132]) have been examined. Almost all of them coincide in similar information about users’ daily life, including sleep metrics (e.g., total sleep duration, number of awakenings), habits’ information (e.g., alcohol and caffeine consumption), and subjective measurements (e.g., sleep quality, restfulness feeling after wake up).

**Figure 11:**
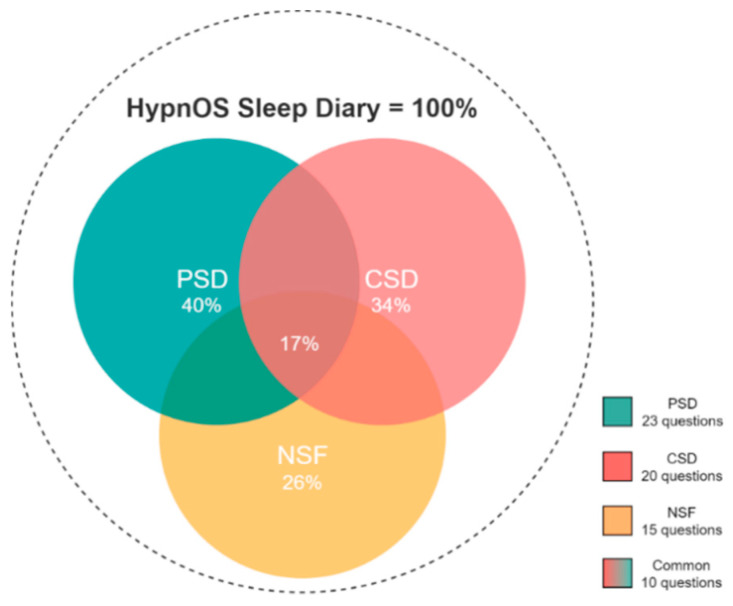
HypnOS Sleep Diary (HSD).

To this end, HypnOS offers a sleep diary, namely the HypnOS Sleep Diary (HSD), which is the superset of the aforementioned diaries, containing the distinct list of questions offered by the three popular diaries, namely PSD, CSD, and NSF (Figure 11). It is worth noting that, despite the fact that HSD contains more questions than the other three sleep diaries, the users are expected to only provide answers to a small set of questions (via the mobile application of HypnOS), since HypnOS has the ability to infer the answers for the remaining enquiries by (i) utilizing contextual information that is offered via the Intelligent Home infrastructure, and (ii) collecting useful data via sleep trackers (Figure 10). The mobile application of HypnOS asks users to complete some questions of the HSD twice on a daily basis: one time in the morning after waking up (post-sleep diary), and one time before going to bed (pre-sleep diary) at night. The questions asked cannot be answered by assessing contextual information and they mainly regard the user’s daily habits (e.g., caffeine and alcohol consumption, smoking), and their subjective perception about sleep (subjective sleep quality, restfulness sense after wake up).

From an end-user perspective, the system presents daily detailed sleep reports, including—amongst others-details regarding sleep patterns, movements during sleep, hours of sleep, etc. HypnOS is able to fuse the collected data (e.g., sleep-related parameters), so as to gain insights about the causes of the residents’ sleep-related issues and act accordingly. More specifically, the mobile application can provide personalized, data-driven sleep recommendations that are based on a collection of sleep hygiene rules (Section 2.6) (e.g., “Drinking coffee two hours before bed time might negatively affect your sleep”), so as to help residents be aware of the potential habits that affect more their sleep quality.

Finally, HypnOS interoperates with the ambient facilities of the Intelligent Bedroom (Figure 1b), so as to adjust a variety of environmental factors (e.g., light, sound), thus enhancing the bedroom environment and creating conditions (Section 2.6.2) that can potentially facilitate the falling asleep and waking up processes. More specifically, in order to improve the waking up process, HypnOS offers a smart alarm that detects the optimal time to wake up residents gently (i.e., when they are in the lightest possible sleep stage) and adjusts bedroom environment according to their preferences (e.g., raise the blinds on alarm ringing). On the other hand, to facilitate the sleep process, HypnOS interoperates with the CaLmi system in order to provide ambient relaxation programs when residents have difficulty in falling asleep.

### 7.2. Architecture

**Figure 12:**
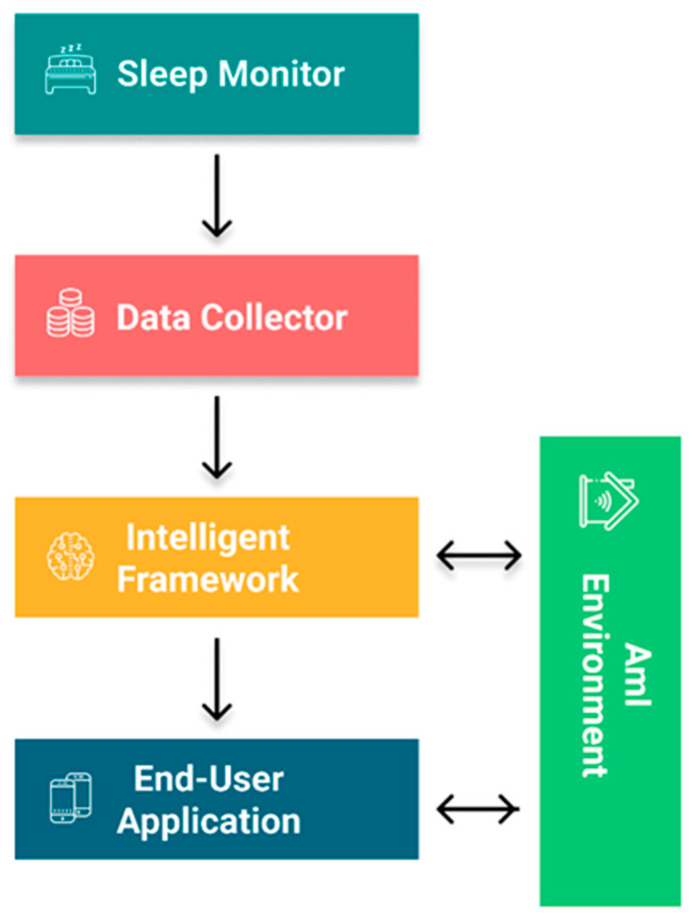
High-level system architecture.

Figure 12 presents a high-level architecture of HypnOS. Firstly, the system through the Sleep Monitor continuously monitors sleep-related data, bio-signals, daily habits and subjective measurements of the residents and informs the Data Collector when new data are available. Next, the data collector aggregates these data, preprocesses them in order to have a consistent format, and finally stores them into a unified data store in the form of a personalized sleep record. Raw data have to be preprocessed to achieve a common format, because they come from different sources and, thus, they are inconsistent. For example, wearable activity trackers record heart rate values every 30 s, while the EEG headband records them every 60 s. To that end, the preprocessor requests these data from the aggregator and makes the appropriate transformations in order to make them consistent. More specifically, the preprocessor harmonizes and normalizes their values, fills-in any missing values (e.g., replaces missing value with the average value of the other sleep trackers), and cleans erroneous data (e.g., repair or delete incomplete data). Afterwards, the Intelligent Framework requests residents’ sleep-related data from the Data Collector and contextual information from the Intelligent Environment and it relies on Artificial Intelligent (AI) to the calculate the daily sleep score of each user (Sleep Score Calculator), generate personalized sleep insights (Sleep Insights Generator), schedule the smart alarm (Smart Alarm Scheduler), and recommend appropriate relaxation programs.

- Sleep Score Calculator predicts the “actual” sleep quality based on the monitored data. In particular, it detects sleep abnormalities in relation to the users’ sleep profile and daily history (e.g., increased bed movements, more awakenings after alcohol consumption) and it outlines their potential causes (e.g., eating a heavy dinner before going to bed). It is based on a heuristic algorithm that takes into account (a) the individual sleep scores that are calculated from the different sleep trackers (e.g., wearable activity tracker/watch), (b) the individual sleep scores that are extracted from the PSQI and SHI sleep questionnaires, which combine different sleep-related parameters (e.g., awakenings, total sleep duration) and other external factors (e.g., drink coffee too close to bedtime, do important work before bedtime), (c) users’ sleep history (average sleep duration, user slept close to his usual time, user woke up easily and close to his usual time), and (d) parameters that are extracted from the pre- and post-sleep diaries (that were provided by the HypnOS mobile application).
- Sleep Insights Generator produces personalized sleep recommendations based on a collection of template sleep hygiene rules. In more detail, it combines sleep data of the previous night with other factors, including: (a) user profile and sleep history information, (b) previous night’s sleep score, and (c) answers to the questions of the sleep diary, to extract personalized insights that encourage inhabitants to adopt healthier routines for the improvement of their sleep quality.
- Smart Alarm Scheduler estimates, the optimal time to wake up residents (e.g., the lightest possible stage of sleep just before desirable wake up time) by exploiting the available data from residents’ sleep profile (e.g., usual wake up time), and daily sleep record (e.g., light sleep). In addition, it takes the wake-up time that the users explicitly have set before they go to sleep into account.
- Sleep Program Recommender suggests relaxation programs for sleep, based on user preferences (e.g., user usually falls asleep with the “Rain Droplets” program) by utilizing information from the user profile. Additionally, it is able to calculate the effectiveness of each program by analyzing information, such as how long the user was in bed trying to sleep, and then adjusts its logic, so as to recommend the most effective programs.

Finally, the processes that take place in the Intelligent Framework result in valuable data that are represented in the end-user application, which also interoperates with the Intelligent Environment.

### 7.3. End-User Applications

The HypnOS system monitors, collects, manages, and creates large amounts of data on a daily basis. Some of those data contain useful information for users (e.g., sleep report, insights) and must be presented to them in an optimal way. Information visualization involves the visual disclosure of information behind the data in a proper display [133]. In order to achieve a usable and pleasant system that is easy to learn, users must understand visualizations in an effortless way and have a natural intuitive mapping of what they represent.

**Figure 13:**
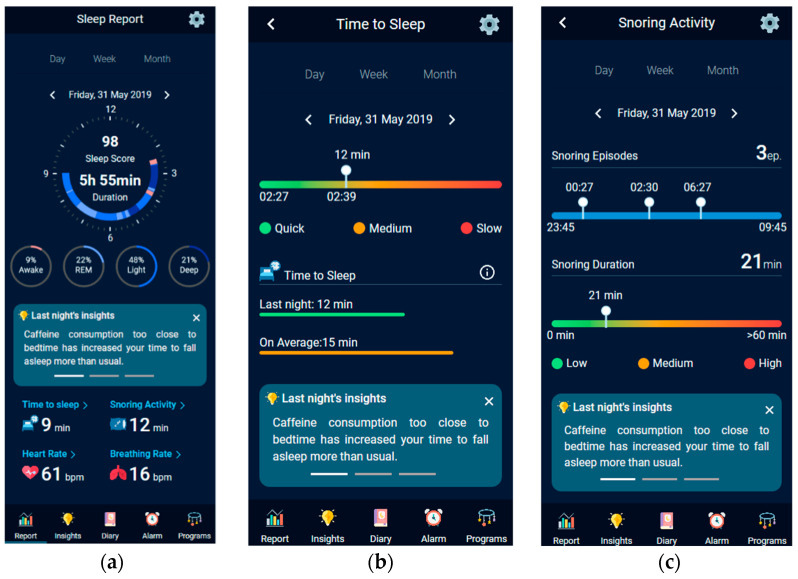
(**a**) Dashboard, (**b**) Time to Sleep, and (**c**) Snoring Activity.

**Figure 14:**
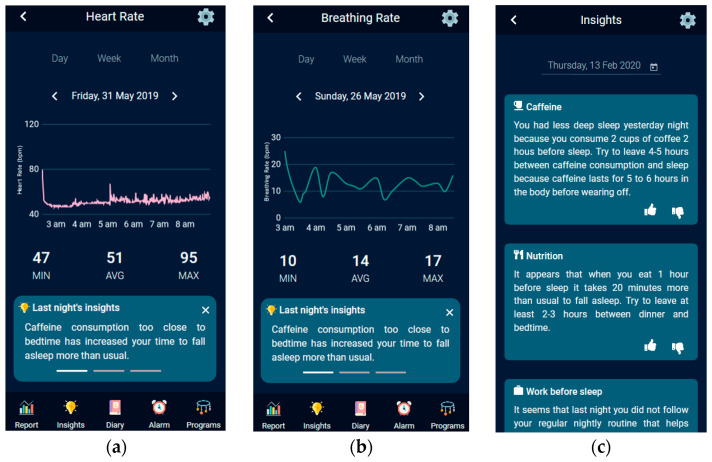
(**a**) Heart rate, (**b**) Breathing Rate, and (**c**) Insights.

**Figure 15:**
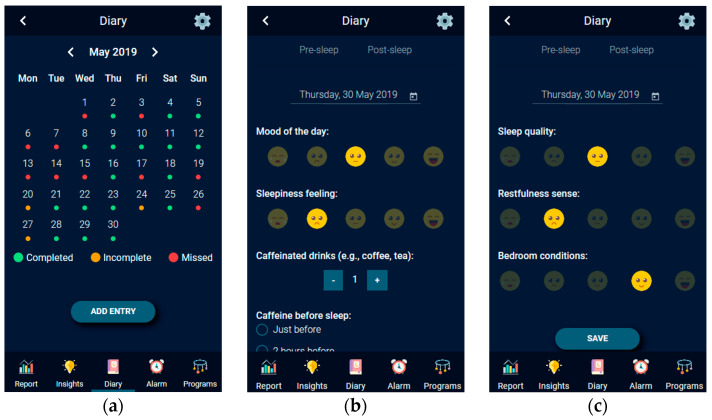
(**a**) Diary, (**b**) Pre-sleep Diary, and (**c**) Post-sleep Diary.

In recent years, mobile devices have become extremely popular, and people believe there should be an app for everything [134]. Using applications, users can access various types of information and control different processes from everywhere and at any time. In particular, with regard to smartphones, according to IDC’s research [135], 79% of owners keep their phone near them for all but two hours of their waking day. Moreover, in the last years, the use of mobile health (m-health), sleep monitoring, and well-being apps has been growing rapidly [136,137,138]. For those reasons, HypnOS provides a user-friendly mobile application that has been developed to facilitate control over the system and assist users. The available functionality is described below:

- Dashboard. Contains a detailed analysis of last night’s sleep (Figure 13a). Users can inspect information regarding (i) their sleep stages (i.e., duration of: Rapid Eye Movement stage, Light Sleep stage, and Deep Sleep stage), (ii) various sleep parameters (e.g., sleep duration, sleep score, time to fall asleep, snoring activity), and (iii) their bio-signals (e.g., nightly heart and breathing rate). Apart from the detailed report, users can find a list of useful sleep insights that they can follow to improve their sleep hygiene.
- Time to Sleep. Presents a horizontal bar chart that displays the time required to fall asleep (Figure 13b). This chart has a gradient fill which consists of three colors (i.e., green, orange, red), representing whether the required time was fast, medium, or slow. Additionally, the users can examine whether this time is in normal range according to their usual time required to fall asleep.
- Snoring activity. Displays two horizontal bar charts representing the user’s snoring episodes during the night, and the total snoring duration (Figure 13c).
- Heart Rate. Presents a spline chart displaying the user’s heart rate activity (Figure 14a), while it also shows the minimum, the maximum, and the average value of the heart rate during the previous night.
- Breathing Rate. Presents a spline chart, which represents the user’s breathing rate activity (Figure 14b). Furthermore, it shows the minimum, the maximum, and the average value of breathing during the previous night.
- Insights. Users are able to view personalized sleep insights on a daily basis. These insights are in the form of recommendations regarding users’ daily habits that affect sleep (e.g., “Drinking coffee two hours before bed time might negatively affect your sleep”). On the top of the page there is a date picker where users can select the date that they want to view their sleep insights. Each insight is associated with a specific category (e.g., Caffeine). Users can state whether they like an insight or not (i.e., upvote or downvote). In this way, this kind of insight does not show up again by clicking the buttons that are on the bottom of the sleep insight’s modal window (Figure 14c).
- Diary. Users are able to view a calendar displaying their diary entries for every day of the current month (Figure 15a). The calendar uses three colors to define the different diary entry states. The red color represents an empty diary entry, the orange represents an incomplete diary entry, and the green represents a successfully completed diary entry. Every day users receive two notification reminders to complete the sleep diary: one after waking up and one before going to bed. In case the users do not wish to fill-in the diary when prompted to do so by the system, they can manually select to do it at any time they wish.
- Pre-sleep Diary. Permits users to answer questions regarding their habits and feelings during the current day. These questions include subjective ratings for mood and sleepiness, as well as activities that may affect the sleep quality, such as caffeine and alcohol consumption (Figure 15b).
- Post-sleep Diary. Permits users to answer questions regarding their personal opinion about previous night’s sleep. These questions include subjective ratings for sleep quality, restfulness sense and bedroom conditions (Figure 15c).
- Alarm. Users can also set an alarm for a specific time, while they can also use the smart wake up feature that is offered by HypnOS. This feature permits users to select a timeframe within which the system must find the best possible moment to wake them up, based on when they are in their lightest stage of sleep. Finally, the users are able to adjust a variety of environmental factors (e.g., lights, sound, blinds, aroma scent), which are going to be activated in the morning, so as to facilitate the waking up process.
- Programs. Users are able to select relaxation programs (e.g., Sound Relaxation, Breathing Relaxation and Guided Imagery) in order to help them sleep. From the quick settings bar, users are able to stop the program at any time and activate or deactivate the ambient light, sound, scent, and projected multimedia.

Finally, the pervasive relaxation player of CaLmi (Section 6.3) became part of the HypnOS system, since it provides the means to create an optimal sleep environment (Section 2.6.2), and offer pervasive relaxation programs (Section 2.6.3) that are appropriate for helping people fall asleep faster. In more detail, exploiting the ambient facilities of the Intelligent Bedroom, the player has the ability to project multimedia to the surroundWall (Figure 1b), play sound and music from the room’s speakers, adjust a room’s lighting conditions, release a pleasant odor into the room using a scent diffuser, etc.

### 7.4. Evaluation

A small-scale formative user-based evaluation experiment was conducted in order to assess the complete functionality of the mobile application of HypnOS and uncover user’s opinion regarding the overall concept of HypnOS, using observation and questionnaires as the main methods. The experiment was conducted in the bedroom simulation space of the Intelligent Home of FORTH-ICS, so as to make the users feel that they are indeed inside a bedroom, and that they use the application before and after going to bed. The main objectives of the user-based evaluation experiment were mainly to identify any unsupported features, uncover potential usability issues, and evaluate users’ overall satisfaction before planning a large-scale experiment.

#### 7.4.1. Participants

A total of eight (8) users participated in the experiment, who were representative of the intended users of the system (adults). This number of users is appropriate for preliminary evaluations, as it can identify important usability problems and eliminate them before proceeding to large scale experiments [139].

**Figure 16:**
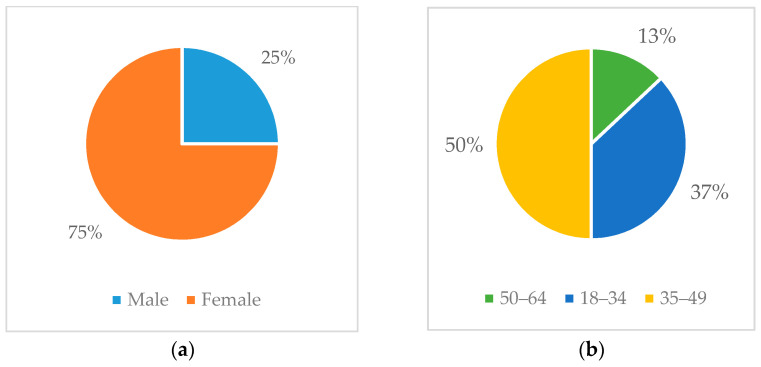
(**a**) Gender distribution and (**b**) age distribution of HypnOS’s evaluation participants.

The selected users meet different characteristics (e.g., age, gender, experience with sleep trackers, or sleep monitoring applications). In particular, from the total of eight users, 75% were females and 25% were males (Figure 16a). Moreover, users were selected in order to include an as wide as possible range of ages, because, according to many studies [140,141,142], sleep patterns change across the lifespan in various ways, including decreases in quantity and quality of sleep. To that end, the participants were stratified into three subgroups (18–34, 35–49, and 50–64 years old) based on their age. In more detail, half of the users (50%) were from 35 to 49 years old, the 37% were from 18 to 34 years old, while the 13% were from 50 to 64 years old (Figure 16b).

None of the participants had experience with sleep monitoring applications or wearable sleep trackers, while the majority of them did not experience sleep difficulties or sleep complaints. In this experiment, there were no specific prerequisites for user participation. However, for future evaluation experiments, we intend to include users who experience sleep difficulties so as to also record their comments and opinion.

#### 7.4.2. Data Collection

A pre-evaluation questionnaire has been used to collect participants’ demographics and other user-related information. Additionally, a custom observation grid was used to collect qualitative and quantitative information, such as the overall duration of the running test, the user comments, the number of users’ errors during each task, the time it took to users to complete each task, and the number of hints provided during each task.

Regarding the post-evaluation questionnaires, the System Usability Scale (SUS) and a custom questionnaire were handed out to the participants in order to reveal the usability of the mobile application, as well as the overall user satisfaction. SUS [143] is a subjective satisfaction questionnaire that includes 10 questions, with five response options for respondents; from strongly agree to strongly disagree, while the custom questionnaire included 13 questions about users’ overall impression regarding HypnOS and its mobile application.

#### 7.4.3. Evaluation Experiment

A facilitator was responsible for orchestrating the entire process and assisting the users when required in order to make participants feel comfortable and ensure that the experiment progressed as planned. In addition, an observer was responsible for recording information in the observation grid, while a member from the experimental team was outside the Intelligent Bedroom, in order to manage any possible technical difficulties.

During the introduction stage, the facilitator welcomed the test participants, gave a brief explanation of the purpose of the experiment, and highlighted the importance of their participation. Next, the concept of the Intelligent Bedroom was described, followed by a short introduction to HypnOS and its mobile application. After the introduction, the participants interacted with the HypnOS mobile application by following the available scenario tasks. The scenario included six (6) tasks, which were given one-by-one to the participants in a presentation-like format, so they could turn to read them again as many times as needed. During the test itself, the facilitator refrained from interacting with the participants. The only exception to this rule was when participants were clearly stuck and were unhappy with the situation. After the test was completed, the facilitator thanked the users for their participation in the experiment, and then asked them to fill in the SUS questionnaire. Finally, the participants were debriefed according to a set of questions, regarding their overall impression for the system, and any other thoughts or suggestions that they had for improvements.

#### 7.4.4. Results

**Figure 17:**
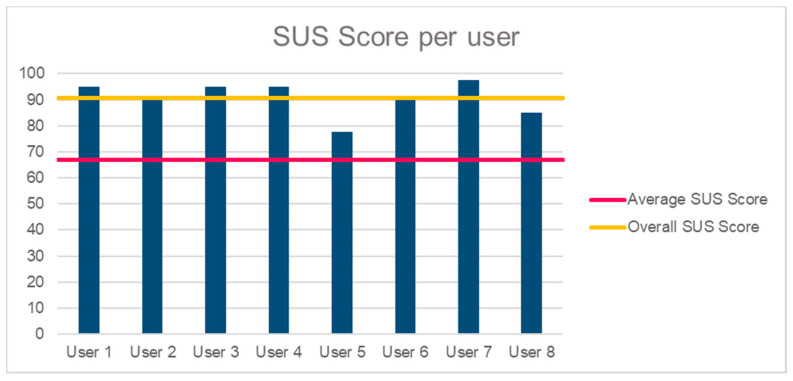
System Usability Scale (SUS) score per user for the mobile application of HypnOS.

The results of this evaluation were very positive, which revealed that users-even first-time ones-can effectively use the mobile application. The overall impression was positive regarding the concept of a system that provides detailed sleep statistics and personalized sleep insights in order to help not only those who have sleep difficulties but practically everybody. In more details, all of the participants successfully completed the six tasks of the given use case scenario. Some minor errors were performed in some cases, which the users were able to overcome either by themselves or by acquiring hints from the facilitator. Furthermore, by analyzing the answers to the SUS questionnaire, it was revealed that the participants’ responses were overall very positive. Figure 17 illustrates the SUS score individually (per user), as well as the overall SUS score for the mobile application of HypnOS. The overall SUS score was 90.63, which is much higher than the average SUS score (68), while, according to the curved grading scale for SUS [144], the system received overall an A+ score.

**Figure 18:**
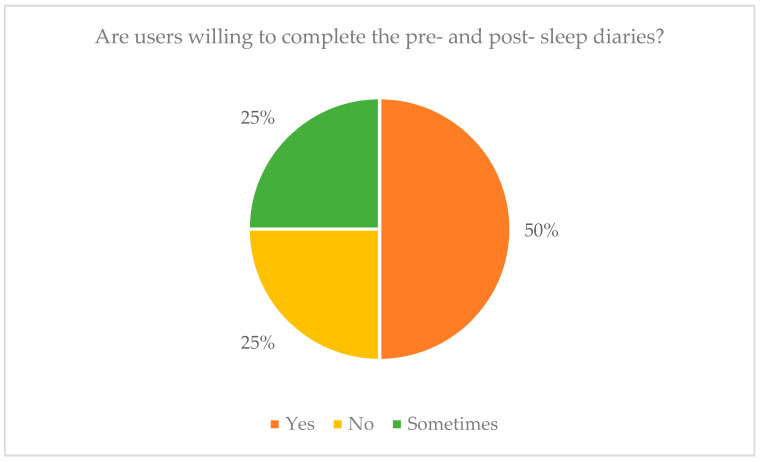
Evaluation participants’ willingness to complete pre- and post- sleep diaries.

It should be noticed that there were some limitations regarding this user experiment. In particular, we would probably receive different user feedback, if the participants had sleep issues. In addition, the experiment was conducted with simulated data, so the insights that were delivered to the users were not personalized to their measurements and daily habits. However, we received useful feedback by observing the users and analyzing the evaluation findings, which will help in designing an improved version of the mobile application from an interaction perspective. During this study, we draw several useful insights:

- Users’ impression of the Intelligent Bedroom was very positive. In particular, there were many positive comments regarding HypnOS’ ability to adjust the environment in order to create appropriate sleep/wake up conditions.
- The concept of HypnOS, although designed for those who have sleep issues, was found to be very useful and helpful for those who do not have any sleep difficulties as well.
- Users did not seem skeptical regarding HypnOS’ ability to collect information about their daily routine and habits, so as to provide personalized sleep insights. On the contrary, the majority of them stated that they do not mind if the system has access to their personal data, provided that they will be safe.
- Most of the users are willing to enter data manually if that means that the system will obtain useful information that result in personalized sleep insights. In more detail, 50% of the participants said that they would be willing to complete pre- and post-sleep diaries on a daily basis, especially if some fields were pre-selected. However, another 25% of participants reported that they would be bored to complete pre-sleep and post-sleep diaries on a daily basis, while the remaining 25% stated that they would complete them sometimes and not on a daily basis (Figure 18).
- The users would be willing to wear/use all the sleep trackers (e.g., wearable activity tracker/watch, under-the-mattress sleep tracker), except from the EEG headband, because they found it very obtrusive and irritating. However, given that individuals facing sleep issues are accustomed to wear various aids while they sleep, we are confident that they will be eager to use the headband provided that they will obtain useful insights regarding their sleep. For example, users that have obstructive sleep apnea and wearing a CPAP machine (a device that sends a steady flow of oxygen into nose and mouth as the wearer sleeps. This keeps the airways open and helps the individual to breathe normally) during sleep may be more willing to use it than others that do not have sleep issues.

## 8. Conclusions and Future Work

The literature confirms that stress interferes with sleep (when considering that insomnia is a common sleep disorder derived from stress [145]), and vice versa, since many report that their stress increases when the length and quality of their sleep decreases [146]. The COVID-19 pandemic has produced unprecedented changes in our lives, generating significant stress, anxiety, and worries about health, social isolation, employment, finances, as well as the challenge of combining work and family obligations [147]. High stress levels and sleep deprivation may lead—among others—to depression, impaired memory, decreased motivation, obesity and cardiac morbidity. To this end, managing and controlling stress, as well as ensuring a good sleep quality, is vital for improving health and the overall quality of life.

Towards this direction, this work has presented two systems that interoperate and aim to assist users that struggle with stress and poor sleep quality. On the one hand, CaLmi is a pervasive stress detection and reduction system for Intelligent Environments, and its ultimate goal is be able to help people relax by enabling the ubiquitous presentation of relaxation programs. It is the first proposed stress management system that uses both biofeedback and contextual information to detect stress and offers pervasive relaxation programs, which use the amenities of the environment to be presented in an ambient and non-intrusive manner. On the other hand, HypnOS is a sleep monitoring and recommendation system that combines various sources of information, giving emphasis to sleep-related parameters, bio-signals and contextual information, so as to provide valuable insights that will help users to improve their sleep hygiene. HypnOS uses the mechanisms of CaLmi so as to help users to fall asleep effortlessly by activating pervasive relaxation programs that are appropriate for sleep.

Both of the systems benefit from the infrastructure of the Intelligent Home, so as to infer information regarding the context of use, the resident’s daily habits, etc. Such information is combined with the real-time data collected by wearable devices so as to understand the status (e.g., stressed, sleep deprived) of the user and offer personalized help. Based on the user studies that we have performed, CaLmi seems to be a promising system for stress management in intelligent environments. The reason is that a relaxation session showed to be more effective and satisfying when the intelligent environment is properly adapted in order to activate different senses, and it is not limited to the visual sense alone. Additionally, users found the concept of HypnOS very useful and helpful not only for those who have sleep issues, but also for those who do not face any sleep difficulties. The added value of HypnOS is also revealed by the fact that it is able to provide useful sleep-related insights without requiring users to fill-in lengthy questionnaires.

When it comes to the discussion of future work, there are some improvements and additions that could be done regarding CaLmi. Firstly, the system could be able to operate with any developed stress detection algorithm. This could be made possible if the developers follow certain predefined coding guidelines. Those guidelines should provide information on the input and output parameters in order for the algorithm to be compatible with the system. Furthermore, an addition could be the support of multiple users in a single relaxation session. That means that two or more users should be able to start a session together, whether or not they are in the same room. Another extension of this work could be the ability to create relaxation programs whose content is not static. In that case, the new relaxation program should be created in steps. At each step, its duration, content, and additional features should be defined. Thus, a relaxation program will represent a series of predetermined steps that are executed one after another in sequence. Finally, another addition could be the introduction of an expert figure (e.g., a psychologist) collaborating with the decision mechanisms in order to better support the user. The expert should be able to monitor the users’ stress levels, create customized relaxation programs for each user, and determine when a relaxation program should be suggested to a specific user.

Regarding HypnOS, future work includes the ability of the system to collaborate with experts. In particular, sleep doctors and sleep experts could have access to sleep data on a 24/7 basis in order to provide better guidance to the users. An expert should be able to monitor users’ sleep behaviors and patterns to enrich the existing sleep hygiene recommendations, determine when a relaxation program should be suggested to a specific user, and advise users to see a doctor in case there are more complex issues (e.g., repeated breathing disturbances). With this in mind, the system could be further expanded in order to have a medical and health care scope.

For both systems, a longitudinal user-based study will be organized to take place, not only to assess the performance and the effectiveness of the systems, but also to fully examine the user experience of living in a home with such ambient facilities.

## Data Availability

Not applicable.
